# Motivators and barriers of seasonal influenza vaccination among primary health care physicians in Qatar

**DOI:** 10.1016/j.pmedr.2024.102595

**Published:** 2024-01-09

**Authors:** Kamran Aziz, Mansoura Ismail, Rizwan Ahmad, Ahmed Sameer AlNuaimi, Marwa Bibars, Muna Mehdar AlSaadi

**Affiliations:** a Primary Health Care Corporation, Doha, Qatar; b Family Medicine, Suez Canal University, Faculty of Medicine, Ismailia, Egypt; c Medical Intern, Faculty of Medicine, Cairo University, Cairo, Egypt

**Keywords:** Influenza, Vaccination, Motivators, Barriers, Physicians, Qatar, Primary care, Seasonal

## Abstract

Annual influenza vaccination is an effective way to reduce the burden of disease throughout the year. A cross-sectional study was conducted in primary healthcare centres in Qatar to determine vaccination coverage among physicians, motivators, and barriers. The vaccination rate was higher among physicians aged 45 years and above (p-value < 0.005). Most primary care physicians (95 %) strongly agree that being vaccinated reduces the risk of disease spread. The most frequently mentioned barriers were the belief that one could still get influenza after being vaccinated and the fear of side effects (92.6 % and 29.5 %, respectively). Health authorities can implement strategies that take these factors into account to increase immunization coverage.

## 1 Introduction

Seasonal influenza causes a burden of disease that persists throughout the year. Illnesses vary in severity, sometimes resulting in hospitalization and death (WHO.int Influenza (Seasonal) (2021)). It is important to get the required vaccination to prevent the spread in healthcare facilities (Morb, 1998, Kapila et al., 1997). and reduce the transmission of influenza from healthcare workers to patients (Carman et al., 2000, Potter et al., 1997).

World Health Organization (WHO) recommends annual influenza vaccination for healthcare workers (Influenza (seasonal) [Internet]. Who.int. (2021)). Studies have also shown that influenza vaccination decreases the overall length of hospital stay for influenza patients (Shot, 2021). If healthcare workers are infected, there is a 30 to 40 % increase in absenteeism which can lead to under-provision of healthcare facilities (Wilde et al., 1999, Saxen and Virtanen, 1999).

Many healthcare workers may remain asymptomatic, leading to a higher rate of infection among patients. This can result in increased morbidity and mortality in high-risk populations (ACIP, 2000). Vaccination among healthcare workers has led to a significant reduction in influenza illness and improved infection rates in patients (Wilde et al., 1999).

There are some misconceptions as catching influenza from vaccination, concerns around pregnancy, and questioning its effectiveness (Eickhoff et al., 2000).

The lack of sufficient information about influenza vaccination among healthcare workers leads to a significant drop in vaccination coverage (Horman et al., 1986).

WHO and Centers for Disease Control and Prevention (CDC) recommend that all healthcare workers should receive an annual influenza vaccine. Vaccinating 80 % of healthcare workers is sufficient to reduce transmission and to ensure herd immunity (Salgado et al., 2004, Influenza Infection Health Risks and Influenza Vaccination Qatar, 2022).

The Primary Health Care Corporation (PHCC) in Qatar provides complimentary influenza vaccines to all PHCC staff members and high-risk patient groups. The target set by the Ministry of Public Health (MOPH) for vaccination coverage of PHCC staff and at-risk patient groups is 65 %. In 2017, the actual coverage was 60 % (Primary Health Care Corporation, 2017).

## 2 Methods

### 2.1 Study design

A cross-sectional study was conducted in all Primary healthcare centres. The online survey was sent to all physicians operating general or family medicine clinics, outpatient clinics or non-communicable disease clinics, or emergency services during October 2021. An invitation email with an introduction to the study and a questionnaire was sent to them. Reminders were sent via email and physician’s WhatsApp group.

### 2.2 Questionnaire

The questionnaire was obtained from a study by Asma et al. (2016) which was validated.

The questionnaire consisted of demographics such as age, gender, years of service in primary care, and influenza-related risk factors. Additionally, questions about influenza vaccination and behavioral factors, which were assessed using five-point Likert questions. Responses were expressed as follows: 1-strongly agree, 2- agree, 3-neutral, 4- disagree and 5- strongly disagree. The main areas of this section were perceived barriers, perceived benefits, and motivating factors.

### 2.3 Statistical analysis

The collected data was analyzed using SPSS version 25. The perceived motivation score was calculated as follows: the percentage reported was for those who answered as agree or strongly agree. Each positive item was awarded a score of one point. The total Motivation Score was calculated by summing the scores of positive items and multiplying it by 100 / maximum count of items. The perceived benefit score was calculated as follows: the percentage reported was for those who answered as agree or strongly agree. Each positive item was awarded a score of one point. The total Perceived Benefit Score was calculated by summing the scores of positive items and multiplying it by 100 / maximum count of items. The perceived barrier score was calculated as follows: The first six items were negatively rephrased to serve the purpose of presenting barriers. Disagree and strongly disagree became equivalent to agree and strongly agree for the rephrased items. The percentage reported was for those who answered as agree or strongly agree. Each positive item was awarded a score of one point. The total Perceived Barrier Score was calculated by summing the scores of positive items and then multiplying it by 100 / maximum count of items.

Bivariate analysis was then carried out using the independent *t*-test for two groups and analysis of variance (ANOVA) for more than two groups to investigate the association between the dependent variables (perceived motivation, benefit, and barrier scores) and the background characteristics of physicians. The level of statistical significance was set at a p-value of less than or equal to 0.05.

## 3 Results

**Fig. 1:**
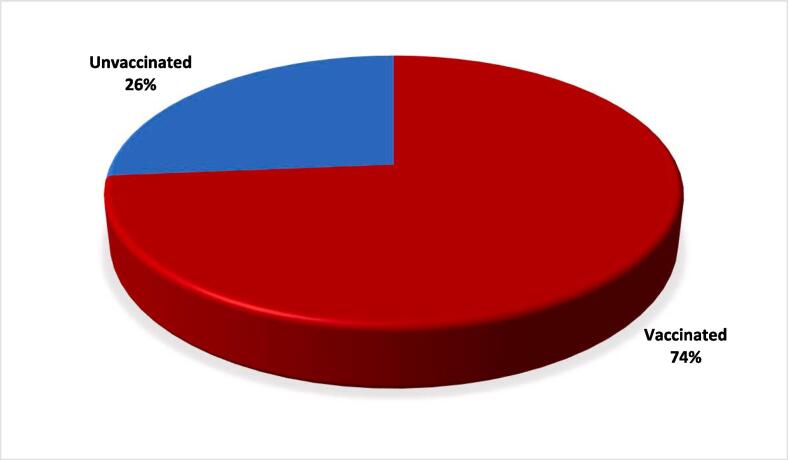
The proportion of vaccinated/unvaccinated primary care physicians in the last season Qatar.

Of 450 questionnaires distributed, 190 completed questionnaires were returned giving an overall response rate of 42 %. Out of 190 physicians,147 (77.3 %) physicians were aged between 35 and 54 years, and more than half of the participants were males. Having chronic diseases was reported in 32.1 % of them. The majority (96.3 %) of participants have been vaccinated against influenza in the past while 73.7 % of physicians had taken influenza vaccine in the last season (Fig. 1).

The vaccination rate was higher among physicians aged 45 years and above, with a 100 % rate among those aged 65 years and above. Such an association was statistically significant (p-value of 0.004). There is a statistically significant association between the vaccination rate, gender, and working experience. Male participants and those with 11 or more years of work experience in primary healthcare had a high rate of vaccination (82.1 % and 86.8 % respectively). Among those having chronic diseases or living with a vulnerable groups such as children aged less than 2 years, people with a chronic disease and the elderly reported high vaccination rates. However, those results were statistically insignificant. Out of 3 physicians living with a pregnant woman, only one was vaccinated. This was not statically insignificant (Table 1).

**Table 1:** Seasonal influenza vaccine uptake according to background characteristics of primary care physicians in Qatar 2021.

Characteristic	Vaccinated physicians (%)	Unvaccinated physicians (%)	N	*p*-Value
Age				
22–34	5(45.5)	6(54.5)	11	0.004^b^
35–44	46(63.0)	27(37.0)	73	
45–54	62(83.8)	12(16.2)	74	
55–64	24(82.8)	5(17.2)	29	
≥65	3(1 0 0)	0(0.0)	3	
Gender				
Male	92(82.1)	20(17.9)	112	0.002^a^
Female	48(61.5)	30(38.5)	78	
Working in PHCC (in years)				
0–3	33(56.9)	25(43.1)	58	0.005^a^
4–7	60(78.9)	16(21.1)	76	
8–10	14(77.8)	4(22.2)	18	
≥11	33(86.8)	5(13.2)	38	
Have chronic disease				
Yes	48(78.7)	13(21.3)	61	0.275^b^
No	92(71.3)	37(28.7)	129	
Living with a child aged < 2 years				
Yes	18(64.3)	10(35.7)	28	0.234^b^
No	122(75.3)	40(24.7)	162	
Living with a person with a chronic disease				
Yes	28(63.6)	16(36.4)	44	0.92^b^
No	112(76.7)	34(23.3)	146	
Living with elderly				
Yes	12(63.2)	7(36.8)	19	0.287^b^
No	128(74.9)	43(25.1)	171	
Living with a pregnant woman				
Yes	1(33.3)	2(66.7)	3	0.141^b^
No	139(74.3)	48(25.7)	187	

a P value is estimated by ANOVA test.

b P value is estimated by independent samples *t*-test.

The respondents rated their level of agreement on a series of statements regarding the motivators and benefits of the seasonal influenza vaccine. Approximately 99 % strongly agree that, MOPH provides seasonal influenza vaccines free of charge for healthcare professionals. In addition, around 96 % and 97 % of them are aware of the MOPH recommendations about influenza vaccine, the specific age group and chronic diseases who should receive vaccination respectively. Regarding the perceived benefits, 95 % strongly agree that being vaccinated reduces the risk of disease spread to their families and patients respectively (Table 2).

**Table 2:** Seasonal influenza vaccine reported motivators and perceived benefits among primary care physicians in Qatar 2021.

	N (Total N = 190)	%	95 % confidence interval
Reported motivator			
			
I know the Ministry of Health's recommendations for influenza vaccination	182	95.8	(92.2–––98)
I know the Ministry of Health recommendations about the age groups and chronic diseases which require influenza vaccination	184	96.8	(93.6–––98.7)
I have sufficient knowledge about influenza	174	91.6	(87–––94.9)
I get information about influenza from reliable sources every year	174	91.6	(87–––94.9)
The Ministry of Health provides free influenza vaccination for health professionals	188	98.9	(96.7–––99.8)
Perceived benefit			
Vaccination reduces the risk of spreading the disease to my patients	178	93.7	(89.6–––96.5)
Vaccination reduces the risk of spreading the disease to my family	181	95.3	(91.5–––97.6)
Community vaccination reduces my workload during an epidemic	174	91.6	(87–––94.9)

**Fig. 2:**
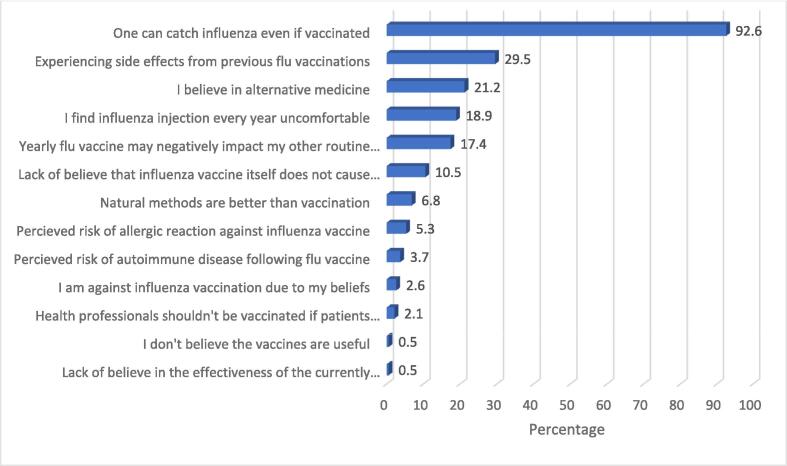
Frequency of perceived barriers among primary care physicians in Qatar 2021.

The most frequent barrier reported by the respondents was their belief that one could get influenza even if vaccinated (92.6 %). This is followed by 29.5 % experiencing side effects from previous influenza vaccinations (Fig. 2).

The mean perceived motivation and benefit scores were higher among those aged 55 years and above, male gender, those with a history of having chronic diseases, current and previous history of influenza vaccine, and those who received 5 doses of influenza vaccine. However, those relationships were statistically insignificant (Table 3 and Table 4).

**Table 3:** Relationship between background characteristics and perceived motivation score among primary care physicians in Qatar 2021.

	Motivation Score					
	Range	Mean	SD	SE	N	P value
Age (years)						0.36^a^
22–34	(68–––100)	82.5	8.1	2.43	11	
35–44	(68–––100)	86.5	9.4	1.11	73	
45–54	(64–––100)	86.9	9.5	1.1	74	
55+	(76–––100)	88.3	8.7	1.53	32	
						
Gender						0.2^b^
Female	(64–––100)	85.7	9.3	1.05	78	
Male	(72–––100)	87.4	9.2	0.87	112	
						
Duration of PHCC work experience in years						0.19^a^
<4	(68–––100)	86.4	9.5	1.24	58	
4–7	(64–––100)	85.5	9.5	1.09	76	
8–10	(76–––100)	90.2	8.5	2	18	
11 and over	(76–––100)	88	8.6	1.4	38	
						
Having chronic disease						0.36^b^
Negative	(68–––100)	86.3	9.3	0.82	129	
Positive	(64–––100)	87.6	9.1	1.17	61	
						
Positive history of influenza vaccination						0.17^b^
Negative	(68–––100)	81.1	9.7	3.67	7	
Positive	(64–––100)	86.9	9.2	0.68	183	
						
Positive history of influenza vaccination last season						0.11^b^
Negative	(64–––100)	84.8	10.3	1.45	50	
Positive	(72–––100)	87.4	8.8	0.75	140	
						
Count of seasonal influenza vaccine doses received-categories						0.1^a^
Never	(68–––100)	81.1	9.7	3.67	7	
(1–2)	(68–––100)	85.8	9.6	1.92	25	
(3–4)	(64–––100)	85	8.9	1.27	49	
5+	(72–––100)	88.5	9.1	0.98	87	

a P value is estimated by ANOVA test.

b P value is estimated by independent samples *t*-test.

**Table 4:** Relationship between background characteristics and perceived benefit score among primary care physicians in Qatar 2021.

	Perceived Benefit Score					
	Range	Mean	SD	SE	N	P value
Age (years)						0.09^a^
22–34	(67–––100)	86.7	11.2	3.36	11	
35–44	(53–––100)	85.3	11.7	1.37	73	
45–54	(67–––100)	90	9.9	1.16	74	
55+	(53–––100)	88.3	12.7	2.24	32	
						
Gender						0.22^b^
Female	(53–––100)	86.5	12.1	1.37	78	
Male	(53–––100)	88.6	10.7	1.01	112	
						
Duration of PHCC work experience in years						0.76^a^
<4	(53–––100)	87.8	11.6	1.52	58	
4–7	(53–––100)	86.8	11.7	1.34	76	
8–10	(73–––100)	88.9	10	2.35	18	
11 and over	(60–––100)	88.9	10.9	1.77	38	
						
Having chronic disease						0.13^b^
Negative	(53–––100)	86.9	11.3	0.99	129	
Positive	(53–––100)	89.5	11.3	1.44	61	
						
Positive history of influenza vaccination						0.17^b^
Negative	(60–––100)	81	11.8	4.47	7	
Positive	(53–––100)	88	11.2	0.83	183	
						
Positive history of influenza vaccination last season						0.1^b^
Negative	(60–––100)	85.3	12.3	1.74	50	
Positive	(53–––100)	88.6	10.9	0.92	140	
						
Count of seasonal influenza vaccine doses received-categories						0.05^a^
Never	(60–––100)	81	11.8	4.47	7	
(1–2)	(53–––100)	83.5	12.2	2.44	25	
(3–4)	(67–––100)	87.1	11.3	1.61	49	
5+	(53–––100)	89.9	10.7	1.14	87	

a P value is estimated by ANOVA test.

b P value is estimated by independent samples *t*-test.

Regarding the mean perceived barrier score and its association with background characteristics of primary care physicians, those not having chronic diseases, those who have not received the influenza vaccine last season, female participants of the study, and those who have never taken the influenza vaccine showed higher scores. Those associations were statistically significant (Table 5).

**Table 5:** Relationship between background characteristics and perceived barrier score among primary care physicians in Qatar 2021.

	Perceived Barrier Score					
	Range	Mean	SD	SE	N	P value
Age (years)						0.61^a^
22–34	(37–––62)	47.4	7.3	2.21	11	
35–44	(26–––69)	46.5	9.1	1.06	73	
45–54	(28–––62)	44.9	7.9	0.92	74	
55+	(28–––63)	45.4	8.7	1.53	32	
						
Gender						0.006^b^
Female	(28–––69)	47.8	8.9	1.01	78	
Male	(26–––65)	44.3	7.8	0.74	112	
						
Duration of PHCC work experience in years						0.77^a^
<4	(26–––65)	45.7	8.5	1.12	58	
4–7	(28–––69)	46	8.4	0.97	76	
8–10	(32–––58)	43.8	6.8	1.6	18	
11 and over	(28–––68)	46.1	9.3	1.51	38	
						
Having chronic disease						0.028^b^
Negative	(28–––69)	46.7	8.2	0.72	129	
Positive	(26–––62)	43.7	8.7	1.11	61	
						
Positive history of influenza vaccination						0.027^b^
Negative	(43–––66)	53.2	7.1	2.68	7	
Positive	(26–––69)	45.4	8.4	0.62	183	
						
Positive history of influenza vaccination last season						<0.001^b^
Negative	(32–––69)	50.6	7.9	1.12	50	
Positive	(26–––68)	44	8	0.67	140	
						
Count of seasonal influenza vaccine doses received-categories						<0.001^a^
Never	(43–––66)	53.2	7.1	2.68	7	
(1–2)	(32–––69)	49.3	9.5	1.89	25	
(3–4)	(28–––63)	47.6	7.6	1.08	49	
5+	(26–––68)	42.8	8	0.85	87	

a P value is estimated by ANOVA test.

b P value is estimated by independent samples *t*-test.

## 4 Discussion

The current study showed that most of the participants were vaccinated against seasonal influenza in the past. Almost three-quarters of physicians were vaccinated in the last season, showing a positive attitude towards influenza vaccination. This is consistent with a study in Saudi Arabia, which showed that around two-thirds of primary care physicians received the vaccine (Alenazi et al., 2018).

Vaccination coverage was higher in the age group 45 years and older. This is consistent with a study in Qatar done by Alhammadi A et al. (Alhammadi et al., 2015). Abu-Gharbieh et al. conducted in three Middle Eastern countries, the highest vaccination uptake was found in the age group > 45 years (32.2 %) in the United Arab Emirates and the age group 36–45 years (69.5 %) in Kuwait. While in Oman, the highest vaccination coverage was found in the age group 36–45 years (56.3 %) (Abu-Gharbieh et al., 2010).

The high vaccination coverage of the current study was also found in people suffering from chronic diseases or living with people who are at risk. However, these results were statistically insignificant. According to a study by Mytton et al. personal protection is an important factor in obtaining an influenza vaccine (Mytton et al., 2013). Alenazi BR et al. reported that healthcare workers understand that because of their interactions with sick patients, they could get influenza and, consequently, their families could be affected (Alenazi et al., 2018).

There was also a statistically significant association between vaccination coverage and work experience. This is consistent with Alenazi BR et al. (Alenazi et al., 2018). One of the factors that led to higher vaccination rates among older participants was a better understanding of the benefits of the vaccine (Bellia et al., 2013, Dini et al., 2017).

According to a study in Saudi Arabia by Haridi et al. awareness of immunization guidelines, longer practice duration, and age > 40 years were independently associated with immunization receipt among healthcare workers (Haridi et al., 2017). The current study showed that the majority of participants agreed that the MOPH provided influenza vaccine free of charge to healthcare workers and were aware of the ministry’s recommendations on the influenza vaccine. They also knew which age groups and chronic diseases should be vaccinated. This was also reported by Alenazi BR et al. where 89 % of healthcare workers were aware of influenza vaccination guidelines (Alenazi et al., 2018).

The majority agreed that vaccination reduces the risk of disease transmission to their families or patients. According to Abu-Gharbieh et al. self-protection was the most common factor influencing the decision to vaccinate (59 %) (Abu-Gharbieh et al., 2010). This is consistent with a study by Haridi et al. which showed that self-protection (81.5 %) was the main reason for vaccination. In comparison, 73.4 % of healthcare workers reported being vaccinated to protect their patients (Haridi et al., 2017). According to a study by Nabil J. Awadalla et al. the most commonly cited motivators for seasonal influenza vaccination were healthcare workers awareness that they are at risk of influenza infection and need protection (77.5 %), the presence of a chronic illness (69.6 %), and a tendency to protect close family members (62.6 %) (Awadalla et al., 2020, Petek and Kamnik-Jug, 2018). Our study showed that motivation scores were higher among those over 55 years and male participants. Alenazi BR et al. stated that male gender, and being a physician significantly increased vaccination compliance (Alenazi et al., 2018).

The most frequently cited barriers by respondents were related to the effectiveness of the vaccine and the belief that one could still get influenza after being vaccinated, followed by side effects from previous influenza vaccines. Other barriers included a belief in alternative medicine and finding the annual influenza injections uncomfortable. Similar results from Abu-Gharbieh et al. found that 24.9 % of respondents did not take the vaccine because they were skeptical about the vaccine's effectiveness. In addition, 20.1 % of healthcare workers were unaware of the protective value of vaccination against disease, while 17.3 % were concerned about the side effects (Abu-Gharbieh et al., 2010). According to a study by Nabil J. Awadalla et al. some of the key barriers among unvaccinated participants were fear of side effects (40 %), a misperception of the unimportance of vaccination (24.1 %), and unsatisfactory prior experience (17.6 %) (Awadalla et al., 2020). Similar results were found in Haridi HK et al. which showed that the reasons for avoiding vaccination were the misconception that the vaccine causes influenza (38.5 %) and concerns about the vaccine's effectiveness (32.7 %) (Haridi et al., 2017).

## 5 Conclusion

The vaccination rate was higher among physicians aged 45 years and over. Most primary care physicians believe that being vaccinated reduces the risk of disease spread. The most frequently mentioned barriers were the belief that one could still get influenza after being vaccinated and the fear of side effects. Strategies that address these factors can increase influenza vaccine uptake in primary care. The free availability of vaccines is an important factor in getting people to be vaccinated. Around one-third of participants think vaccination should be mandatory for healthcare workers.

## 6 Limitations of the study

This study has some limitations. The vaccination receipt was self-reported and was not confirmed by any vaccination records. This could lead to an overestimation of vaccination coverage. There might be a possibility of information recall bias, however, the survey was conducted for three weeks in October 2021 during flu season.

Statement of human rights

All procedures performed in studies involving human participants were in accordance with the ethical standards of the institutional and/or national research committee and with the 1964 Helsinki declaration and its later amendments or comparable ethical standards.

Ethics approval

The Institutional Review Board at PHCC in Qatar approved this study (Reference number PHCC/DCR/ 2020/06/047).

## Funding

Open Access funding provided by the Qatar National Library.

## CRediT authorship contribution statement

**Kamran Aziz:** Conceptualization, Methodology, Writing – original draft, Writing – review & editing. **Mansoura Ismail:** Writing – review & editing, Methodology. **Rizwan Ahmad:** Writing – review & editing. **Ahmed Sameer AlNuaimi:** Formal analysis, Data curation. **Marwa Bibars:** Formal analysis, Data curation. **Muna Mehdar AlSaadi:** Writing – review & editing.

## Declaration of competing interest

The authors declare that they have no known competing financial interests or personal relationships that could have appeared to influence the work reported in this paper.

## Data Availability

The authors do not have permission to share data.
